# Bayesian Networks Predict Neuronal Transdifferentiation

**DOI:** 10.1534/g3.118.200401

**Published:** 2018-05-30

**Authors:** Richard I. Ainsworth, Rizi Ai, Bo Ding, Nan Li, Kai Zhang, Wei Wang

**Affiliations:** * Department of Chemistry and Biochemistry; † Graduate Program in Bioinformatics and Systems Biology, University of California, San Diego, La Jolla, California 92093-0359

**Keywords:** systems biology, gene regulation network, neuroscience

## Abstract

We employ the language of Bayesian networks to systematically construct gene-regulation topologies from deep-sequencing single-nucleus RNA-Seq data for human neurons. From the perspective of the cell-state potential landscape, we identify attractors that correspond closely to different neuron subtypes. Attractors are also recovered for cell states from an independent data set confirming our models accurate description of global genetic regulations across differing cell types of the neocortex (not included in the training data). Our model recovers experimentally confirmed genetic regulations and community analysis reveals genetic associations in common pathways. Via a comprehensive scan of all theoretical three-gene perturbations of gene knockout and overexpression, we discover novel neuronal *trans*-differrentiation recipes (including perturbations of SATB2, GAD1, POU6F2 and ADARB2) for excitatory projection neuron and inhibitory interneuron subtypes.

The classification of cortical neurons is a debated topic with differing schemes using anatomical, molecular and physiological characteristics in order to make distinctions. It is generally accepted that there exist two major groups of neurons namely, excitatory Projection Neurons (PNs) (Greig *et al.* 2013) and inhibitory Interneurons (INs) (Kepecs and Fishell 2014). All neurons are generated only during embryonic development (Lodato *et al.* 2015) after which class-specific traits remain unchanged for the life of the organism. It is classically thought this precludes any change in identity postnatally. Intriguingly neurons may exhibit more plasticity than previously thought. As far back as 2002, astrocytes were directly reprogrammed into neurons and more recently post-mitotic neurons have been converted from one subtype to another in young animals as reviewed by Amamoto *et al.* (Amamoto and Arlotta 2014).

A Bayesian network (BN) is a graph-based model of joint multivariate probability distributions that captures properties of conditional independence between variables. Bayesian networks can be used for representing statistical dependencies in a set of data and were applied to the problem of reconstructing gene regulation networks (GRN) from expression data by Friedmann *et al.* (Friedman *et al.* 2000) and Hartemink *et al.* (Hartemink *et al.* 2001). It is known that the protein transcription factor produced by one gene can have a causal effect on the expression of another gene. BN can be used to represent the conditional dependencies between genes and thus interpret these as causal patterns of gene regulations.

We challenge the paradigm that neurons of the mammalian cortex are a permanently post-mitotic and differentiated cell type via modeling genetic perturbations that facilitate direct transdifferentiation. In this theoretical study we present the application of Bayesian network techniques to high quality deep-sequencing data in order to reverse engineer the genetic regulations in human neurons. We identify those attractors that correspond to different neuron subtypes and validate our model with an independent data set. Using dynamic bayesian inference we derive interconversion recipes between differing neuron subtypes and from the perspective of the cell-state potential energy landscape, describe those interconversion pathways.

## Method

### Data processing, clustering and discretization

**Figure 1:**
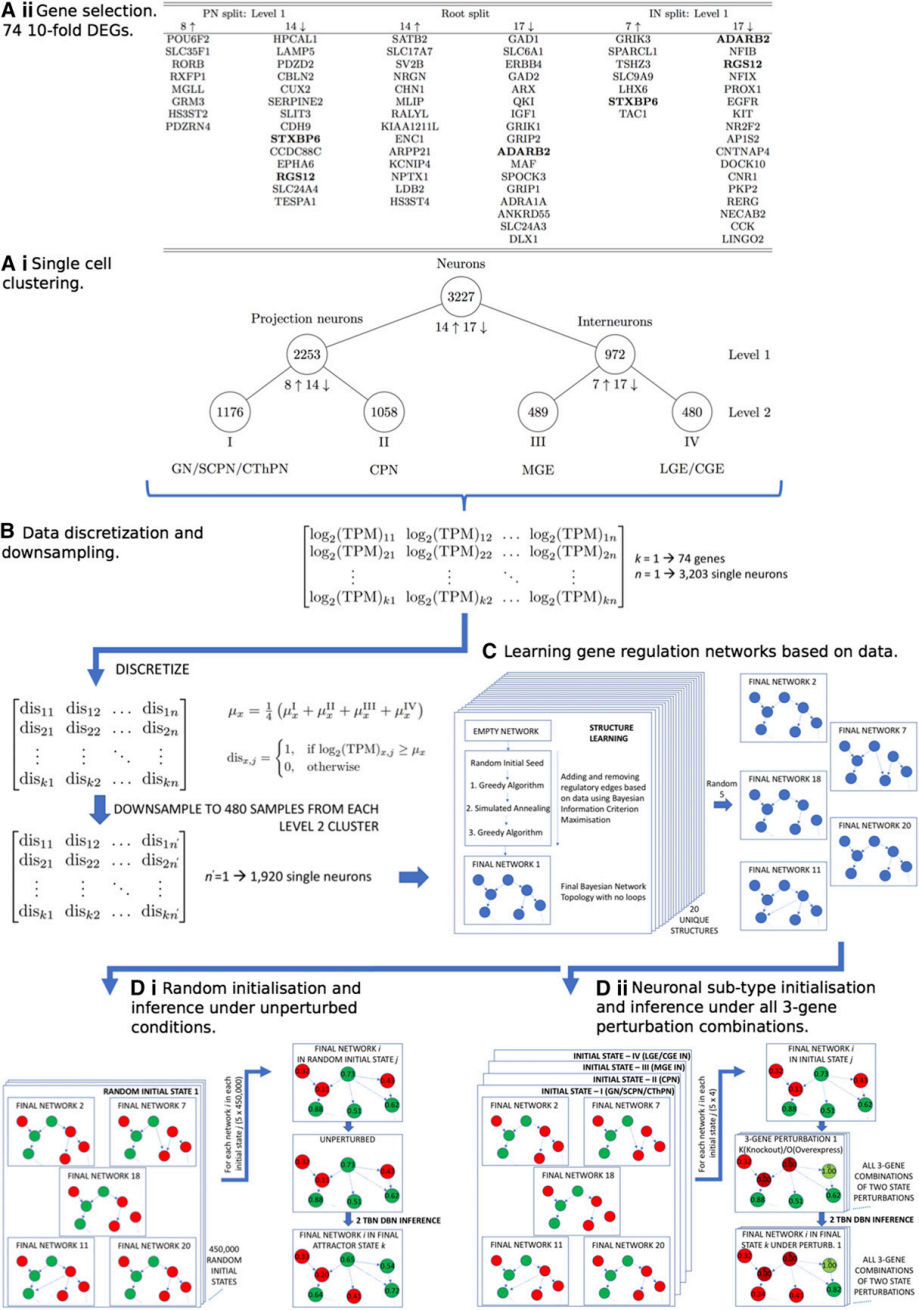
Workflow pipeline. A.ii. Hierarchical clustering of 3,227 quality-filtered single neuron data sets from previous single-nucleus RNA sequencing study. Number of 10-fold up-(↑) and down-regulated (↓) DEGs given at each junction. These are defined as up-regulated in the subsequent left hand branch and down-regulated in the left hand branch each relative to the right hand branch. Terminal clusters I - IV at Level 2 used in this work.(Lake *et al.* 2016). A.i. 77 ten-fold DEGs (of which 74 are unique) used to train networks. Splits refer to junctions in the hierachical tree in Figure 1 A.ii. Genes in bold occur in multiple splits. B. Data discretization on the weighted arithmetic mean of the log_2_(TPM) for each gene across 3,203 Level 2 single neurons. Data downsampled to 1,920 samples. C. Structure learning directed acyclic gene reulation networks using the discretized downsampled data with local and global optimization routines. 20 random seeds used to generate 20 different structures. 5 structures randomly chosen for further calculations. D.i. 450,000 random initialisations of the nodes in the continuous interval [0,1] for the 5 network structures. 2 TBN DBN inference performed for each network in each initial state. Converged attractor states subsequently clustered. D.ii. Nodes in 5 network structures initialised in the continuous interval [0,1] corresponding to the four neuronal subtype cell states. For each network structure in each initial state, 2 TBN DBN inference carried out for all 3-gene perturbation combinations (clamping nodes as overexpressed or knocked out for duration of inference). Subsequent node probabilities averaged over the 5 structures for each 3-gene perturbation in each initial state.

**Figure 2:**
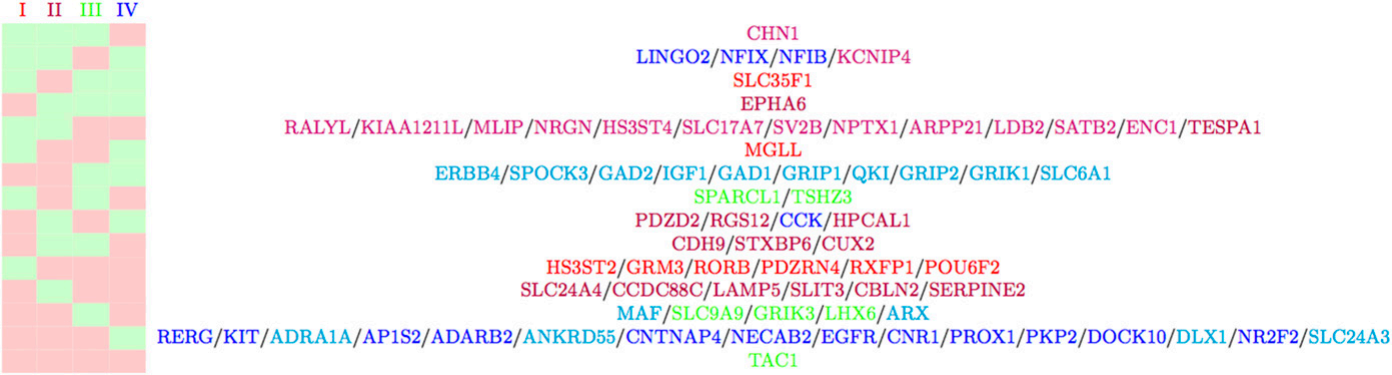
Experimental barcodes for clusters I - IV post data processing. For gene expression probabilities ≥0.5 code displayed as green and for gene expression probabilities <0.5 code displayed as red. Genes that display the same pattern across all four clusters are displayed on the same row. Genes are colored red, purple, green and blue for those expressed in clusters I - IV respectively. DEG that are expressed in both PN subtypes I and II are colored magenta and DEG expressed in both IN subtypes III and IV are colored cyan.

Lake *et al.* (Lake *et al.* 2016) previously conducted single-nucleus RNA sequencing on post-mortem adult human cerebral cortex and generated 3,227 quality-filtered single neuron data sets. These nuclei were subsequently resolved into 17 clusters, based on the differential regulation of 16,242 protein-coding genes, through repeated rounds of unsupervised hierarchical clustering and supervised classification (technical details can be found in ref. (Lake *et al.* 2016)). Figure 1 A.i. is a reproduction of the authors hierarchical tree down to Level 2 including the clusters considered in this work. At each of the 3 splits, we consider the ten-fold differentially expressed genes (DEGs) giving a total of 74 unique genes. Transcription levels were previously analyzed as $\log_{2}$ of transcript per million mapped reads (TPM). Thus, for the complete data set at Level 2 (1176+1058+489+480=3,203 samples) we calculate the weighted arithmetic mean for each gene $\mu_{x}=1/4\left(\mu_{x}^{\text{I}}+\mu_{x}^{\text{II}}+\mu_{x}^{\text{III}}+\mu_{x}^{\text{IV}}\right),$ where $\mu_{x}^{\text{cluster}}=1/n\sum_{j=1}^{n}\log_{2}{\left(\text{TPM}\right)}_{x,j},$ where *n* is the number of samples in each cluster. Each data point was subsequently discretized according to:

$${\text{dis}}_{x,j}=\{\begin{array}{ll} 1, & \text{if}\log_{2}{\left(\text{TPM}\right)}_{x,j}\geq \mu_{x}\\ 0, & \text{otherwise} \end{array}$$ (1)

thus transforming the data:

$$\begin{array}{c} \left[\begin{array}{cccc} \log_{2}{\left(\text{TPM}\right)}_{11} & \log_{2}{\left(\text{TPM}\right)}_{12} & \ldots & \log_{2}{\left(\text{TPM}\right)}_{1n}\\ \log_{2}{\left(\text{TPM}\right)}_{21} & \log_{2}{\left(\text{TPM}\right)}_{22} & \ldots & \log_{2}{\left(\text{TPM}\right)}_{2n}\\ \vdots & \vdots & \ddots & \vdots \\ \log_{2}{\left(\text{TPM}\right)}_{k1} & \log_{2}{\left(\text{TPM}\right)}_{k2} & \ldots & \log_{2}{\left(\text{TPM}\right)}_{kn} \end{array}\right]\\ ↓\\ \left[\begin{array}{cccc} {\text{dis}}_{11} & {\text{dis}}_{12} & \ldots & {\text{dis}}_{1n}\\ {\text{dis}}_{21} & {\text{dis}}_{22} & \ldots & {\text{dis}}_{2n}\\ \vdots & \vdots & \ddots & \vdots \\ {\text{dis}}_{k1} & {\text{dis}}_{k2} & \ldots & {\text{dis}}_{kn} \end{array}\right] \end{array}$$

where gene *x* runs from 1 to k=74 and sample *j* runs from 1 to n=3,203. The experimental barcodes for each cluster, post-discretization, are shown in Figure 2.

In order to prevent the network topologies being biased toward any one neuronal sub-type, the discretized data were down-sampled via the random removal of samples from clusters I, II and III until each cluster contained 480 samples, thus matching the lowest cluster size, that of cluster IV. For each of the three downsampled clusters, the appropriate number of samples were randomly selected for removal and the probability of expression (assigned according to the fraction of the 480 samples that were in a state 1 post-discretization) was calculated for each gene. Comparison was then made to the probability of expression for each gene from the complete, non-downsampled cluster via the root mean square deviation (RMSD) and Pearson R correlation summed over all 74 genes. This process was repeated $2\times 10^{4}$ times for each cluster and the set of samples that gave the lowest resultant RMSD were removed. The RMSD/Pearson R values for each downsampled cluster compared to the relevant complete cluster were I:0.0279/0.9976, II:0.0239/0.9986 and III:0.0451/0.9891. This gave a data matrix of size 1920×74 for 20 separate structure learning runs.

### Structure learning

For a given directed acyclic graph (DAG) model G based on data *D* with *n* binomial variables, it can be shown that

$$P\left(D|G\right)=\prod_{i=1}^{n}\prod_{j=1}^{q_{i}}\frac{\Gamma \left(N_{ij}\right)}{\Gamma \left(N_{ij}+M_{ij}\right)}\frac{\Gamma \left(a_{ij}+s_{ij}\right)\Gamma \left(b_{ij}+t_{ij}\right)}{\Gamma \left(a_{ij}\right)\Gamma \left(b_{ij}\right)},$$ (2)

where *n* is the number of variables, $q_{i}$ is the number of instantiations of the parents $X_{i},$ $a_{ij}$ is the ascertained prior belief of the number of times $X_{i}$ takes its first value when parents $X_{i}$ are in their jth instantiation, $b_{ij}$ is equivalent to $a_{ij}$ but with $X_{i}$ taking its second value, $N_{ij}=a_{ij}+b_{ij},$ $s_{ij}$ is the number of times in the data $X_{i}$ takes its first value when parents $X_{i}$ are in their jth instantiation, $t_{ij}$ is equivalent to $s_{ij}$ but with $X_{i}$ taking its second value, $M_{ij}=s_{ij}+t_{ij}$ and Γ(x) is the gamma function (Neapolitan 2009). Equation 2 is defined as the Bayesian score assuming Dirichlet priors. In order to punish overly complex DAGs and reduce the possibility of overfitting, we use the Bayesian information criterion (BIC) to score structures:

$$BIC\left(G:D\right)=\text{ln}\left(P\left(D|G\right)\right)-\frac{d}{2}\ln m,$$ (3)

which includes an error term, where *m* is the number of samples and *d* is the dimension of the DAG *i.e.*, the number of parameters.

Our procedure follows a three-stage score-based approach common to the method of Chang *et al.* (Chang *et al.* 2011) but omitting prior knowledge incorporation and is thus purely data driven. We give a brief overview here for the readers convenience. Since the problem of learning optimal structure is NP-hard (Chickering *et al.* 1995) we use heuristics in the form of the greedy algorithm to maximimise the BIC score during the first stage. Starting with an empty network, two random nodes (genes) A and B are selected. If no edge exists between them, either the directed edge A → B or the opposite regulation B → A is generated, each with a probability of 0.5. If an edge already exists between the two nodes (a possibility from the second step onwards), it is either reversed or deleted each with a 0.5 probability. For each of the four outcomes, the change to the network is accepted if the BIC score increases, else it is rejected. In our case, this started with an empty network and was iterated $2.5\times 10^{4}$ times (1/40 NSteps).

**Figure 3:**
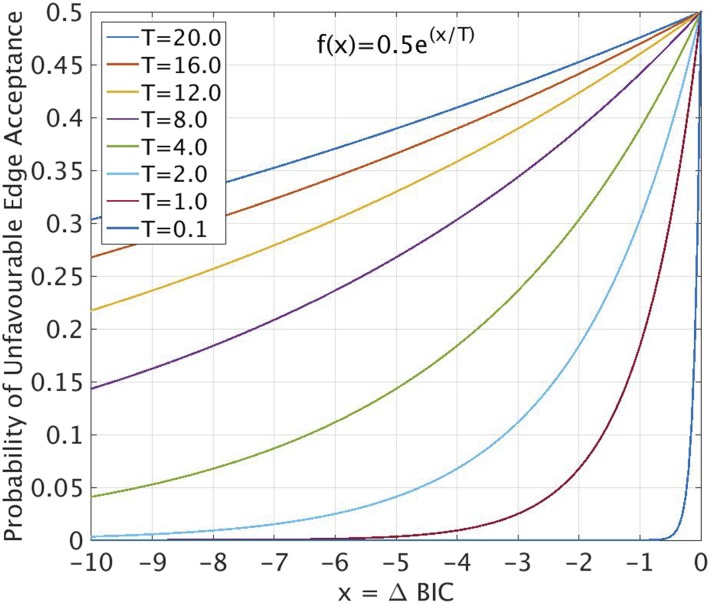
Acceptance criterion for edges that are unfavorable to BIC score during simulated annealing stage of structure learning. Representative temperatures in the range 0.1≤T≤20 plotted.

During the second stage, we employ the metaheuristic approach of simulated annealing in order to approximate the global minimum. The network is instantaneously heated to a temperature T and uniformly cooled over the course of the stage. According to the logic set out in stage 1, an edge is generated. However, if this leads to an unfavorable decrease in the BIC score, the edge is accepted with a probability of $\text{P}\left(\text{edge}\;\text{accepted}\right)=\text{1}/2{\text{e}}^{-\Delta BIC/T}.$ Figure 3 shows this probability as a function of the ΔBIC for a given edge introduction, at different temperatures. Stage 2 was iterated $1\times 10^{6}$ times (NSteps) from a starting T=20 down to T=0.

**Figure 4:**
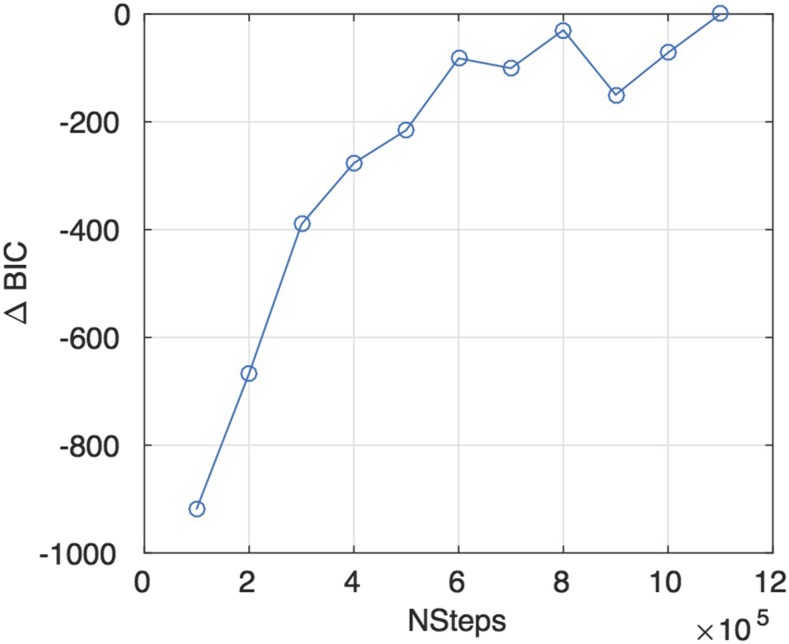
Convergence of ΔBIC (referenced from structure with 1.1M Nsteps) with NSteps (defined as number of steps during simulated annealing phase). Each value is the arithmetic mean over 5 independent structure runs.

Stage 3 consists of a final stage BIC maximization via the greedy algorithm, identical to the protocol of stage 1. This was iterated $4\times 10^{4}$ times (1/25 NSteps). The change in the average BIC score was converged with the value of NSteps as shown in Figure 4.

Since the data used was non-temporal, only edges that did not introduce a loop into the structure were accepted. We employed this structure learning procedure using 20 random seeds and learnt an ensemble of models.

### Inference

During inference we apply Bayes’ rules to obtain the posterior probabilities. Since we are interested in developing a gene regulation model that can be initialised into neuronal subtype cell states; for each source cluster I - IV (see Figure 1 A.i.), initial probabilities for each node *x* were defined as the probability that the node was in a state 1, *i.e.*, expressed, for the given cluster and were input as values in the continuous interval [0,1]. These probabilities were assigned according to the fraction of the total samples, in the given cluster, that where in a state 1 post-discretization.

### Model averaging

We use the full Bayesian framework and do not attempt to approximate one true underlying distribution with a single structure. The *a posteriori* distribution of models is:

$$\begin{array}{c} P\left(G|D\right)=\frac{P\left(D|G\right)P\left(G\right)}{P\left(D\right)}\\ \propto P\left(D|G\right)P\left(G\right), \end{array}$$ (4)

where P(D|G) is the likelihood of the model and the prior, P(G), is not assumed to be uniformly distributed and thus not constant. We therefore perform probabilistic inference by model averaging where the averaged conditional distribution of variable *X* is obtained by integrating over models:

$$\begin{array}{c} P\left(X|D\right)=\int P\left(X|G\right)P\left(G|D\right)dG\\ \propto \int P\left(X|G\right)P\left(D|G\right)P\left(G\right)dG. \end{array}$$ (5)

Since the model space is written as $M=\left\{\left(s_{k},{\tilde{\theta }}_{k}\right),k=1,\ldots ,K\right\},$ where ${\tilde{\theta }}_{k}$ is the continuous ensemble of the conditional probability table (CPT) configurations for each structure $s_{k}.$ For every $s_{k},$ each possible parameterization in the CPT configuration ensemble $\theta \in {\tilde{\theta }}_{k}$ defines a member, $m=\left\{\left(s_{k},\theta \right),k=1,\ldots ,K\right\}$ and the distribution is normalized against all models:

$$P\left(m\right)=P\left(s_{k},\theta \right)=\frac{P\left(\theta |s_{k}\right)P\left(s_{k}\right)}{\alpha },$$ (6)

where the normalization factor $\alpha =\sum_{k^{\prime }=1}^{K}P\left(s_{k^{\prime }}\right)\int_{\theta }P\left(\theta |s_{k^{\prime }}\right)d\theta .$ Equation 5 is thus extended to a double-integral over structure space and structure-dependent parameter space:

$$\begin{array}{c} P\left(X|D\right)\propto \int \int P\left(X|m\right)P\left(D|m\right)P\left(s,\theta \right)dsd\theta \\ \propto \sum_{k=1}^{K}\int_{\theta }P\left(X|s_{k},\theta \right)P\left(D|s_{k},\theta \right)P\left(s_{k},\theta \right)d\theta \\ \propto \sum_{k=1}^{K}\int_{\theta }P\left(X|s_{k},\theta \right)P\left(D|s_{k},\theta \right)\frac{P\left(\theta |s_{k}\right)P\left(s_{k}\right)}{\alpha }d\theta . \end{array}$$ (7)

The Bayesian network models *k* sum over discrete structure space and the parameter vector configuration *θ* integrates over continuous parameter space and can thus become intractable by analytical methods. Within the Markov Chain Monte Carlo (MCMC) approach we use the order statistics of a uniform distribution [0,1] to simulate a sample from posterior distribution $P\left(\theta |s_{k}\right)$ (a beta-density function).

### Learned parameter retrieval

For a given structure, the probability of gene *X* with no parents remains as the initial probability for the course of the inference run. For gene *X* with one parent gene *Y*, the probability of expression of gene *X* (*i.e.* P(X=1)) is dependent on the two parameters $\theta_{1}^{X}=P\left(X=1|Y=1\right)$ and $\theta_{2}^{X}=P\left(X=1|Y=0\right)$ which represent the two respective conditional probabilities. We calculate these conditional probabilities according to the associated beta-density function as:

$$\theta_{1}^{X}=\text{beta}\left(y_{1}x_{1}+1,y_{1}x_{0}+1\right)$$ (8)

and

$$\theta_{2}^{X}=\text{beta}\left(y_{0}x_{1}+1,y_{0}x_{0}+1\right),$$ (9)

where $y_{h}x_{k}$ are the number of data samples where *Y* and *X* occur with the values *h* and *k* in the discretization (h,k={0,1}). The additive value of 1 is for the case that there are no data samples in the given instantiation. A gene X with multiple parents $\pi^{X}$ has $2^{pa\left(X\right)}$ associated with it, where pa(X) is the number of parents of gene *X*. Thus the more general formulation for the parameters is:

$$\theta_{j}^{X}=\text{beta}\left(\pi_{j}^{X}x_{1}+1,\pi_{j}^{X}x_{0}+1\right),$$ (10)

where $\pi_{j}^{X}x_{k}$ is the number of samples in the data where the parents of *X* occur collectively in state *j* and *X* occurs in state *k* (k={0,1}).

### Dynamic Bayesian network

To simulate the evolution of cell state from initial state to the equilibrium or steady state solution we use the dynamic Bayesian network (DBN) model. Here the probabilistic inference is performed by using a 2-time slice Bayesian network (2TBN) and the interface algorithm, (Murphy 2002) which uses static junction trees as a subroutine to compute exact inference in the 2TBN which is then repeated sequentially over time. Accordingly node (gene) probabilities (expressions) evolve over time according to:

$$P\left(X=1;t\right)=\sum_{j}P\left(X=1|\pi^{X}=j\right)P\left(\pi^{X}=j;t-1\right),$$ (11)

where P(π=j) is the product of the probabilities of each parent of *X* being in the binary state {0,1} corresponding to the collective state *j*. Thus the probability of *X* being expressed at time *t* depends on its parameters, $\theta_{j}^{X},$ as well as the probabilities of expression of its parents $\pi^{X}$ at time (t−1). Each set of parameter samples *θ* forms an instance of the DBN model which were averaged for each inference run. The inference runs for each structure $s_{k}$ were then averaged for the final result. During perturbations, node combinations were either clamped at P(X=1)=1 (overexpression) or P(X=1)=0 (knockout) for the duration of the inference run.

### Landscape analysis

In order to identify those attractors in the unperturbed state, we randomly initialise the genes in the continuous interval [0,1] and perform 2TBN DBN. The resulting method corresponds to converging on the minima accessible to the region of cell state space in which the initialisation took place. As per the method of inference under perturbation, node probabilities evolve over time according to Equation 11. In the case of attractor identification we do not apply any clamps and all nodes are free to evolve to a steady state solution. For all inference calculations a set of parameter samples *θ* forms an instance which are averaged over for each unique random initialisation. This process was repeated 450,000 times until the number of unique attractors had converged (234 attractors defined in binary cell state space) thus the sample is large enough to accurately describe the entire space. The basin size of the potential energy landscape corresponding to each attractor can then be calculated as a percentage of the total number of samples that converge to each state.

### Transition states

Transition states during inference were calculated as per Chang *et al.* (Chang *et al.* 2011) by applying a maximum-a-posterior (MAP) estimation to predict the state-transition pathways. That is, at each time step t, the state which maximizes the cell state posterior at the current time step is selected as the current cell state:

$${\hat{S}}_{t}=\arg\max_{\forall S\in S_{t}}\left(P\left(S_{t}\right)\right),$$ (12)

where the probability propagation in DBN cell states is defined as:

$$P\left(S_{t}\right)=\sum_{S_{t-1}}P\left(S_{t}|S_{t-1}\right)P\left(S_{t-1}\right).$$ (13)

For each unique binarised cell state, the state probability for the *i*th state ($P\left(S_{i}\right)$) and thus the potential energy, $U_{i}=-\ln\left(P\left(S_{i}\right)\right)$ of the state *i* can be calculated. These potential energy values for all $\text{M}=2^{74}$ binary states can be represented as $\bar{U}=\left\{U_{1},U_{2},\ldots ,U_{\text{M}}\right\}.$ Cell potentials differ according to cell state conditions. In this work we investigate the cell state potential changes for specific transition states under a specific 3-gene perturbation $\bar{U}|E_{\text{perturb}}.$

### Method validation

Buganim *et al.* (Buganim *et al.* 2012) have previously conducted a single-cell gene-expression analysis of mouse embryonic fibroblasts (MEFs) during cellular reprogramming. They profiled 48 genes from early time points, intermediate cells, and fully re-programmed iPSCs. These data were used to train a simplified Bayes model of hierarchical gene regulation in iPSCs. Using their regulation topology they chose five transcription factor combinations predicted to induce activation of the pluripotency circuitry and generate fully reprogrammed iPSCs. These were experimentally verified via flow cytomeric analysis using OCT4-GFP with ≥0.2% reprogramming efficiency. Using this independent dataset we learnt 20 independent structures and performed inference calculations to predict the effect of their experimentally verified three and four gene overexpression combinations using our methods. The reference Pearson R correlation between the MEF initial cell-state and the fully reprogrammed iPSc final state was calculated to be 0.155. The average Pearson R correlation of the experimentally verified reprogramming recipes and the final iPSc cell-state was predicted to be 0.838 using our methods. This was compared to 20 random 3-gene overexpression combinations with an average Pearson R correlation of 0.270.

### Data availability

Data used has been previously deposited with dbGaP (accession phs000833.v3.p1), curated by the NIH Single Cell Analysis Program Transcriptome (SCAP-T) Project (http://www.scap-t.org) as stated in Lake *et al.*(Lake *et al.* 2016). Supplemental material available at Figshare: https://doi.org/10.25387/g3.6349553.

## Results And Discussion

### Experimental expression profiles

Lake *et al.* (Lake *et al.* 2016) categorized excitatory PN by layer position and as such, cluster I neurons (which were further split in their hierachical tree) were labeled as a combination of granular neurons (GN) from layer 4, sub-cortical projection neurons (SCPNs) from layer 5 and cortico-thalamic projection neurons (CThPNs) from layer 6. Cluster II were classified as cortical projection neurons (CPNs) residing in layers 2/3. The CPNs were shown to express CPN-associated CUX2 and the layer 2/3 marker gene LAMP5 both of which were 10-fold DEGs between clusters I and II and thus included as nodes in our network. Functionally, layers 1-3, termed the supragranular layers, are unique in the neocortex and are the primary origin and termination of intracortical connections. These can be functionally contrasted with internal granular layer 4 and infragranular layers 5 and 6. RORB a marker for layer 4 neurons (Schaeren-Wiemers *et al.* 1997) is also shown to be up-regulated in cluster I compared with cluster II consistent with this analysis. Interneuron subcategories were found to be distributed across across the neocortex and were classified based on developmental origin. (Lake *et al.* 2016) Cluster IV IN were found to originate from lateral (LGE), or caudal ganglionic eminences (CGE) and were VIP+ and RELN+ with positive expression of P8 and NR2F2. Whereas cluster III IN showed MGE marker expression such as LHX6 and SATB1.

The 74 unique genes used to train the GRNs in this work are given in Figure 1 A.ii. They are those that are 10-fold DEGs between the clusters at Levels 1 and 2 of the hierarchical tree (see Figure 1 A.i.). The retrieval of expression profiles for clusters I - IV post data processing and discretization was conducted via the generation of experimental barcodes (see Figure 2). For each cluster, the gene probabilities were calculated as outlined in the Methods section and subsequently binarised based on a cutoff of 0.5. In this way each cluster is represented as one of the $2^{74}$ possible states. As a further assessment of the clustering and cluster uniqueness, we compared the RMSD and Pearson R correlation, summed over all node probabilities in [0,1], between all four clusters (see Table I). From this we retrieve the fact that inter-neuron expression differences for the excitatory PN clusters I and II are less distinct (${\text{RMSD}}^{\text{I vs}\;\text{II}}=0.3358$ and $\text{Pearson}\;{\text{R}}^{\text{I}\;\text{vs}\;\text{II}}=0.5853$) than those between the IN clusters III and IV (${\text{RMSD}}^{\text{III}\;\text{vs}\;\text{IV}}=0.3832$ and $\text{Pearson}\;{\text{R}}^{\text{III}\;\text{vs}\;\text{IV}}=0.2390$) in agreement with Lake *et al.* (Lake *et al.* 2016). For all other intra-neuron subtype comparisons (*i.e.*, PN sub-categories *vs.* IN sub-categories) we find negative correlations exist in the range −0.2805 to −0.5156, thus find, with the possible exception of comparison between clusters I and II, the cluster expression profiles to be adequately unique (within our state space defined by the 74 DEGs) after data processing and discretization.

**TABLE I:** Experimental RMSD/Pearson R correlation, summed over all 74 node probabilities, between clusters I - IV

	II	III	IV
I	0.3358/0.5853	0.4884/-0.2805	0.5704/-0.5077
II	—	0.5655/-0.4221	0.6125/-0.5156
III	—	—	0.3832/0.2390

### Landscape analysis

**Figure 5:**
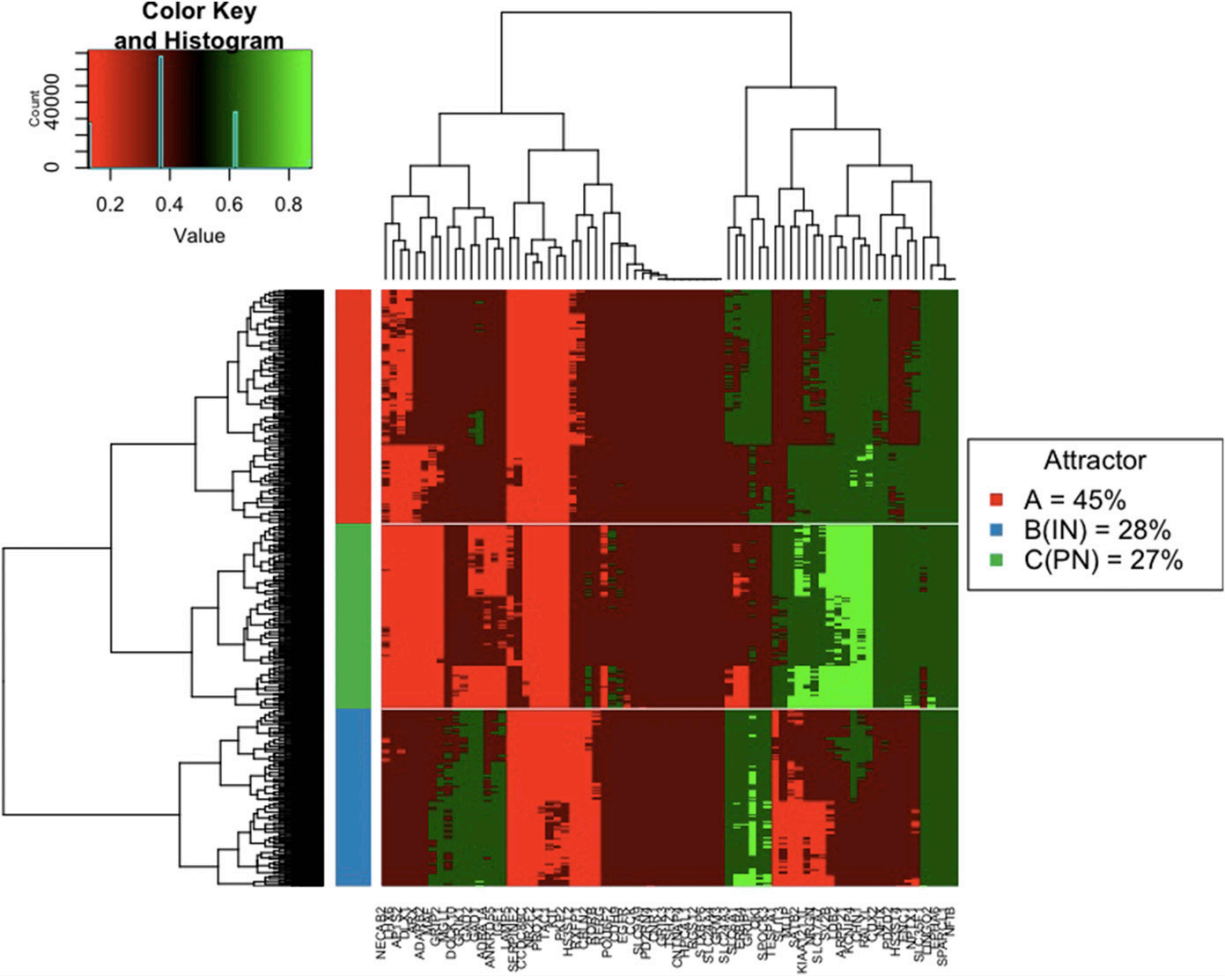
Attractor heat map for all 1082 unique attractors of the unperturbed network model. Cell states defined using quaternary intervals 0.00−0.25,0.25−0.50,0.50−0.75,0.75−1.00 and hierarchically clustered using the heatmap.2 function in R. Basin sizes for the 3 representative cell state clusters A, B and C given in legend.

Due to the computational intensity of 2TBN DBN inference over a sufficiently large sample of state space, five structures were randomly chosen, from the ensemble of 20 BN structures that were learnt, for landscape analysis and inference calculations. For attractor analysis node probabilities for the 74 genes (1 single cell state) were randomly initialised in the continuous interval [0,1] and the node probabilities were converged to a steady state. Post ensemble averaging the node probabilities were discretized. The process was repeated for more randomly selected initial cell states until the number of unique attractors (local minima) was converged. We found that the random sampling of 450,000 initial states was sufficient to identify all the major attractors in the network. 1082 unique attractors were found and hierarchically clustered using the heatmap.2 function with the Euclidean distance metric in R as shown in Figure 5. Arbitrarily cutting the tree at a distance of 1.3, groups the attractors into 3 representative cell state clusters. The basin size of a given attractor on the cell state potential landscape can be defined as the percentage of random initial cell states that converge to the given attractor. The basin size for each unique attractor within each of the three representative cell state clusters A, B or C was summed to give the total basin size for those representative state clusters.

An important test of the four models was to assess the correlation between the initial experimental gene expression values (calculated as described in the Method Section) and those expression values (node probabilities) to which the structure relaxes during 2TBN DBN inference in the unperturbed state. In terms of the cell-state potential landscape, this represents the proximity of the nearest (defined in terms of cell state similarity) “local” minimum or attractor to which the inference results converge. All Pearson R rank correlation coefficients between initial experimental values and relaxed unperturbed inference results are given in Table II. All values are in the range 0.72≤Pearson R≤0.90 showing that the model adequately describes the neuronal cell states corresponding to subcategories I–IV.

**TABLE II:** Pearson R rank correlation coefficients between initial cluster gene expression values and relaxed state node (gene) probabilities in the unperturbed state when initialised in experimental state

Cluster	Pearson R
I	0.8996
II	0.8701
III	0.8055
IV	0.7232

The two unique attractors corresponding to the two PN subcategories I and II cluster together in representative cell state cluster C (see Figure 5) with a basin size covering 27% of cell state space. Further, the two unique attractors corresponding to the two IN subcategories III and IV cluster together in representative cell state cluster B with a basin size covering 28% of cell state space.

In order to further validate our network model, additional single-cell RNA-Seq samples, taken from further Brodmann areas in the adult human cerebral cortex and processed in identical fashion to Lake *et al.* (Lake *et al.* 2016), were used. These samples included neuronal and non-neuronal single cells, such as glia, astrocytes, oligodendrocytes and microglia. Subsequent sample filtering resulted in 546 quality samples which were discretized in the binary interval [0,1] based on the arithmetic mean for each gene summed over all samples. This resulted in all 546 samples displaying a unique cell state (using the same 74 gene space as the model). To make comparison to the attractors predicted by the network model, the 1082 quaternary cell states (clustered in Figure 5) were binarised resulting in 234 unique attractors. Comparison between the additional samples and network attractors was made with the Hamming distance metric. It was found that 451 of the 546 additional samples (83%) had a hamming distance of 20 or less with one or more of the 234 network attractors, *i.e.*, 54 or more genes (73%+) were in the same state of expression. This is suggestive of the fact that the network model (trained only on neuronal subtypes) captures global genetic regulations for other non-neuronal cell types of the mammalian cerebral cortex in addition to accurately describing neuronal cell states.

### Topological analysis

As previously described, 20 BN structures were trained from the combined down-sampled data of clusters I - IV (see Figure 1). The mean number of edges learnt across all 20 structures was $\mu_{\text{edges}}=224.85$ and the standard deviation $\sigma_{\text{edges}}=2.41.$ The relative sparsity of these networks ($\text{D}=\mu_{\text{edges}}/{\text{E}}_{\max}=0.042$) owes to the inclusion of the error term in the BIC scoring function (see Equation 3) leading to the penalisation of overly complex structures. (We use, in the standard definition of network density (D), the number of possible edges in a complete graph (Emax = n(n − 1)) in a directed network where we do not allow self-regulation/loops but hypothetically cycles would be included (a formulation forbidden in our BN learning approach).) A consequence of this and learning multiple structures is that despite the highly stochastic nature of the learning protocol, edges that do occur in higher frequencies across structure learning runs should represent true biological regulations that are coded for in the data.

### Community Detection & Pathway Analysis

**Figure 6:**
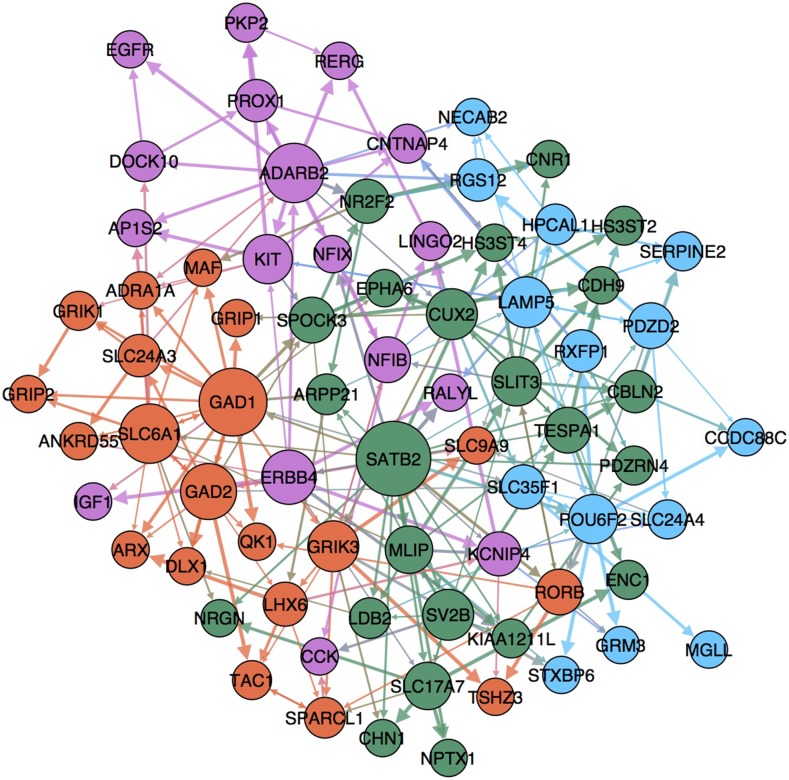
Merged GRN for 20 independent BN structures. 228 unique edges with frequency >0.40 included (from a total of 870 unique edges summed over all 20 BN). Displayed using Yifan Hu algorithm as implemented in Gephi version 0.9.1. Node size proportional to out-degree and edge width proportional to frequency. Community detection algorithm (Blondel *et al.* 2008) run with resolution of 1.55. Modularity = 0.421. Number of communities = 4.

Figure 6 shows the merged GRN for all 20 structures with only those edges that occur in 40% of structures displayed. Node sizes are scaled by out-degree. Community detection analysis using the fast unfolding heuristic algorithm of Blondel *et al.* (Blondel *et al.* 2008) shows there to be 4 communities at a resolution of 1.55. Interestingly of the 20 nodes depicted as being members of the orange community 13 (65%) were also up-regulated IN relative to PN and as such were DEGs from the root split, furthermore 6 (30%) of genes in this community were further up-regulated in cluster III relative to cluster IV from the IN split. This suggests that regulatory mechanisms in this community likely lead to strong co-expression and activating regulation between the nodes in IN and more specifically cluster III IN (MGE derived). Furthermore 13 of the 16 genes (81%) in the purple community are up-regulated in cluster IV IN (LGE/CGE derived) relative to cluster III IN. The same analysis on the green community reveals that 12/23 (52%) genes are broadly up-regulated in PN at the root split and 6/23 (26%) are further up-regulated in cluster II CPN. Finally, the blue community is almost exclusively, with the exception of two nodes, from the PN split with 5 and 7 genes up- and down-regulated respectively in the I GN/SCPN/CThPN cluster. Based on the fact that the network gene list derives from IN and PN specific DEGs, the neuronal subtype DEG specificity, as related to the communities, is partially expected.

Pathway analysis of all 74 genes reveals a significant number of enriched pathways from “Signal Transduction””, “Immune System”, “Transmembrane transport of small molecules” and “Neuronal System” among others. In particular within the “Neuronal System”, the “Neurotransmitter release cycle” (*P* value $=4.36\times 10^{-3}$) includes the genes GAD1, GAD2, SLC6A1 and SLC17A7, the first 3 of which are part of the orange community and form a clique (complete subnetwork with edges between all 3 members in both directions) each forming an edge with each other at a frequency of >0.40 across all 20 structures. The pathway “Signaling by Type 1 Insulin-like Growth Factor 1 Receptor” (*P* value $=5.81\times 10^{-3}$) containing the genes IGF1, ERBB4, KIT and EGFR is also enriched. All of these genes are identified by community analysis to be part of the same community and further, the two edges ERBB4→IGF1 and ERBB4→KIT occur in 100% and 50% of all structures respectively.

### Edge distribution

The top 27 edges that occur in all 20 structures learnt are given in Table III. One of these regulations is SLC17A7→CHN1. SLC17A7, a known regulator of brain physiology, is a brain-specific solute carrier and is found to regulate the neuronal signal transducer, chimerin-1 (CHN1). SLC17A7 specifically functions as as glutamate transporter and it has been found that α1-chimerin regulates dendritic spine density. (Van de Ven 2005) Spine morphological changes, associated with long-term depression, can be induced in hippocampal neurons by metabotropic glutamate receptor activity suggesting possible support for this learnt regulation.

**TABLE III:** Top 27 edges that are learnt in all 20 structures

SLC17A7→CHN1
ERBB4→IGF1
ADARB2→KIT
NR2F2→CNR1
GRIK3→SLC9A9
CUX2→EPHA6
PROX1→PKP2
ADARB2→RERG
POU6F2→STXBP6
LHX6→ARX
SATB2→RALYL
POU6F2→GRM3
SPOCK3→HS3ST4
SLIT3→CDH9
SLIT3→HS3ST4
GAD1→DLX1
NFIB→NFIX
TESPA1→CDH9
ADARB2→EGFR
GAD2→TAC1
SLC17A7→ENC1
SPOCK3→CDH9
KIT→PKP2
SLC17A7→NPTX1
GAD1→QKI
GRIK3→TSHZ3
CBLN2→SERPINE2

The POU family members are transcriptional regulators, many of which are known to control cell type-specific differentiation pathways. STXBP6 codes for the syntaxin binding protein 6 (amisyn) and an edge that occurs in all structures is POU6F2→STXBP6. STXBP6 is known to regulate SNARE complex assembly, a protein complex involved in membrane fusion, that play an important role in neurotransmitter release.

ADARB2 forms two edges that occur in all structures and is the rank 3 node as ranked by total degree (see Table IV) suggesting it plays an important role in neuronal gene regulation and identity. It is a member of the ADAR family which contains 3 members, two of which, ADAR and ADAR1, are catalytically active. The ADARB2 gene encodes a catalytically inactive protein, expressed in brain, amygdala and thalamus. (Hogg *et al.* 2011) It is known to prevent the binding of other ADAR enzymes to targets *in vitro*, and decreases the efficiency of these enzymes. These enzymes are responsible for RNA editing via the conversion of adenosine to idenosine which has been observed in some pri-miRNAs(Kawahara *et al.* 2007; Yang *et al.* 2006); that can in turn affect the function of miRNAs which are thought to have a functional roles in gene regulation. The edge between ADARB2→EGFR occurs in all structures. Interestingly EGFR/MAPK has been shown to regulate AGO2, (Adams *et al.* 2009) which itself is a member of the AGO protein family that play a central role in the function of the RNA-induced silencing complex (RISC) and therefore potentially miRNA function. (Höck *et al.* 2007)

**TABLE IV:** Top 20 nodes as ranked by degree summed over all 20 structures. Average degree per structure given in parentheses

GAD1 (18.20)
SATB2 (18.15)
ADARB2 (16.40)
SLC6A1 (14.30)
ERBB4 (14.20)
GAD2 (11.20)
GRIK3 (11.00)
POU6F2 (10.90)
CUX2 (10.55)
SV2B (10.35)
LAMP5 (9.50)
MLIP (9.45)
SLC17A7 (9.30)
SLIT3 (9.20)
PDZD2 (8.65)
NFIB (8.50)
SPOCK3 (8.45)
KIT (8.30)
TESPA1 (8.10)
LHX6 (7.90)

NFIB, a gene essential for brain development in mice (Steele-Perkins *et al.* 2005) and NFIX form another high frequency edge NFIB→NFIX (see Table III). Both genes belong to the NFI family encoding site-specific transcription factors whose functional diversity is generated in part through protein heterodimerization, (Liu *et al.* 1997) thus providing strong evidence for a protein-protein interaction and a mechanism of co-regulation.

The edge LINGO2→RERG occurs in 85% of structures and is part of the purple community found to be up-regulated in cluster IV LGE/CGE derived IN. Putative homologs of these genes were found interacting in other organisms such as the protein-protein binding interaction in *Drosophila melanogaster* of CG31692 and ics and in saccharomyces cerevisiae the protein-protein interaction between RAS2 and CYR1.

### Transdifferentiation gene recipes

Throughout this section we refer to the “source state” as node probabilities that are initialised to the given probability of expression for the source cluster I–IV and the “target state” as the node probabilities of the final or target cluster I–IV.

Perturbations were applied as either overexpression (clamping the node probability to 1 for the course of inference) or knockout (clamping the node probability to 0 for the course of inference). A full scan of the three-gene recipe combinatorial space was conducted. The calculations were performed using five randomly selected structures from the 20 trained and final node probabilities were averaged over these structures under each perturbation. Three-gene recipes for the 12 interconversions were ranked based on the RMSD between all node probabilities for non-perturbed genes (71) in the perturbed source state post relaxation and the corresponding node probabilities of the target state. The five best recipes for each of the 12 interconversions are given in Supplementary Tables I and II.

Table V shows the best 12 interconversion recipes. We find symmetries exist between the recipes, for example with the exception of S-II→T-I, for conversion to PN subtypes I and II the overexpression (↑) of SATB2 is in all recipes (irrespective of source cluster type). Contrastingly, all of the best 6 conversion recipes to IN subtypes III and IV include the knockout (↓) of SATB2. SATB2 is a DNA-binding protein that regulates chromatin organization and gene expression and is important in the development of corticocortical connectivity in the developing cerebral cortex in mice. (Alcamo *et al.* 2008) Broadly defined as an excitatory marker, SATB2 was found to be regulated between the PN and IN and is a DEG at the root split (see Figure 1 A.ii.). Topologically, SATB2 is the rank-two gene by degree (see Table IV) forming 18.15 edges on average per structure and further occupies a central position in the network making connections to all 4 communities (see Figure 6). The SATB2 protein through its interactions with both the CTIP2 promoter upstream region and histone deacetylase complex, controls chromatin remodeling. Upper layer pyramidal neurons lose their identity in the absence of SATB2 (Britanova *et al.* 2008) perhaps consistent with our prediction of SATB2 knockout being important in inter-neuron PN → IN transdifferentiation and also a regulation that is required to be “held in place” for inter-neuron IN subcategory → IN subcategory transdifferentiation. Similarly, but in reverse regulation logic to SATB2, the knockout of GAD1 is in all but one of the 6 recipes for conversion to PN subtypes and its overexpression is in 50% of the recipes for conversion to IN subtypes namely, S-I→T-III, S-II→T-III and S-II→T-IV. GAD1 is an inhibitory marker and is up-regulated in IN. It is the rank one node by degree forming 18.20 edges on average per structure and connects to nodes from three of the four communities identified (see Figure 6). GAD1 is involved in pathways including “Neurotransmitter release cycle” and “Transmission across Chemical Synapses” and is an integral enzyme in “Gaba Synthesis”. The overexpression of GAD1 is consistent with transdifferentiation to IN targets since the majority of IN are GABAergic.

**Table V.** The 12 best three-gene recipes between source (S-) and target (T-) clusters as ranked by RMSD. Perturbations defined as overexpressed ↑ (node clamped to probability of 1 during inference) and knockout ↓ (node clamped to probability of 0 during inference)

	T-I	T-II	T-III	T-IV
		TESPA1↑	ADARB2↓	ERBB4↑
S-I	–	POU6F2↓	GAD1↑	ADARB2↑
		SATB2↑	SATB2↓	SATB2↓
	GAD1↓		ADARB2↓	ADARB2↑
S-II	TESPA1↓	–	GAD1↑	GAD1↑
	POU6F2↑		SATB2↓	SATB2↓
	GAD1↓	GAD1↓		NFIB↑
S-III	POU6F2↑	POU6F2↓	–	ADARB2↑
	SATB2↑	SATB2↑		SATB2↓
	GAD1↓	GAD1↓	ADARB2↓	
S-IV	POU6F2↑	POU6F2↓	GRIK3↑	–
	SATB2↑	SATB2↑	SATB2↓	

In addition to these inter-neuron transdifferentiation recipe symmetries there also exist symmetries in targeting specific PN or IN subtypes. For example, the best 3 recipes targeting T-I include the overexpression of POU6F2 and by contrast all the best 3 recipes to T-II include the knockout of POU6F2. POU6F2 is a transcription factor involved in DNA binding and is only expressed in the CNS. Moreover the gene is enriched in GO terms for “central nervous system development” and “regulation of transcription, DNA-templated” consistent with its high rank by degree and the fact that it forms edges as a parent node to nodes in three communities. Finally of note in the best transdifferentiation recipes (see Table V) is ADARB2, of which the knockout and overexpression targets cluster III and cluster IV IN respectively. This is the rank three node by degree and is the most connected gene in the purple community in Figure 6 and its functions were discussed in the Edge Distribution subsection.

**Figure 7:**
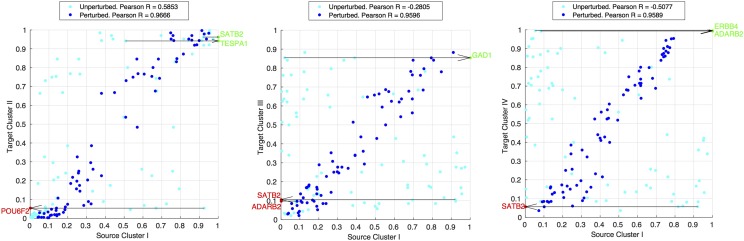
The 3 best transdifferentiaion recipes as ranked by RMSD between source cluster I perturbed states and experimental target states. Subplots are target state node probabilities *vs.* source state node probabilities in the unperturbed (experimental *vs.* experimental) and perturbed (target experimental *vs.* source theoretical perturbed) states, represented by cyan and dark blue data points respectively. Best recipes are highlighted in green (overexpression clamp) and red (knockout clamp) and arrows are drawn to show the direction and magnitude of node probability for the given clamp.

Figure 7 shows representative plots for the three best 3-gene recipes from source cluster S-I. Each scatter plot contains the target state node probabilities *vs.* first the source state node probabilities in the unperturbed (experimental *vs.* experimental) states in cyan and second the perturbed (target experimental *vs.* source theoretical perturbed) states in dark blue. We can see remarkable reprogramming success as reflected by the improvement in Pearson R correlation coefficient which in the case of S-I→T-IV changes from a strong negative correlation in the experimental *vs.* experimental plot of −0.5077 to 0.9589 under the perturbation ERBB4↑ / ADARB2↑ / SATB2↓. All the 12 best transdifferentiation recipes achieve final correlations in the range 0.9351≤PearsonR≤0.9812 (see Supplementary Tables I and II).

### Transition state analysis

**Figure 8:**
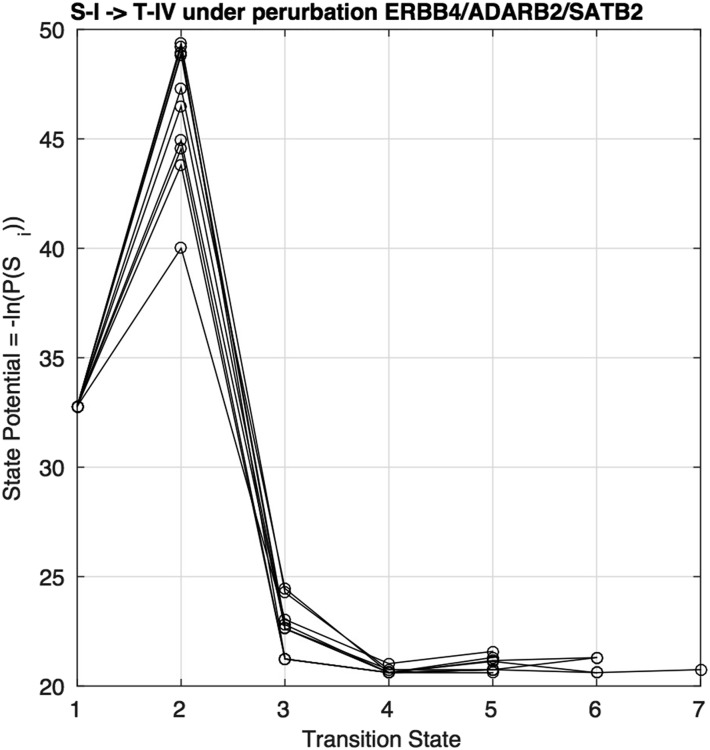
Cell state potential changes along a representative path for a single structure under perturbed conditions. Each line represents a different sampling of the conditional probabilities. The best 3-gene recipe for the interconversion between S-I→ T-IV, ERBB4↑ / ADARB2↑ / SATB2↓, is shown.

It is instructive to monitor cell state probabilities during the transdifferentiation procedure via node clamping. In terms of the cell state potential energy landscape, each of the $2^{74}$ cell states (as represented by a unique combination of 74 binarised node states) in the unperturbed landscape has a potential energy associated with it that is calculated as $U_{i}=-\ln\left(P\left(S_{i}\right)\right),$ where ($P\left(S_{i}\right)$) is the state probability for the *i*th state. Under a 3-gene perturbation the available cell states with a finite probability is reduced by a factor 1/8 to $2.36\times 10^{21}$ and the probability of remaining states also changes. This adjustment to the landscape results in the re-positioning of minima and of energy barriers on the landscape which effectively makes states which were previously inaccessible, open to sampling. Figure 8 shows the probability of the states along the transition paths for 10 independent runs for the transdifferentiation S-I→T-IV under the perturbation ERBB4↑ / ADARB2↑ / SATB2↓ for one of the five structures used in inference. The initial unperturbed state (labeled transition state 1) has a potential energy of 33, the perturbation is then applied which raises the energy of the system to 40−49 “transporting” the cell to a new, previously inaccessible area on the potential energy landscape. This new energy allows the cell to now relax to the new minima which coincides with the target cluster T-IV. We can see that the probability is converged in 4−6 transition states which is common for most recipes.

### Conclusions

In this work, we applied Bayesian network methods to make *de novo* predictions for neuronal transdifferentiation recipes between Projection neuron and Interneuron subtypes. Our network, trained on high quality single-cell RNA-Seq data, accurately describes the four cell subtypes in the unperturbed state and is well validated against an additional data set of single-cells from more varied areas of the human cerebral cortex, using attractor analysis. Many of the regulatory edges learnt between the genes are validated from the wider literature and community analysis reveals significant enrichment in neuron specific pathways among others. We conducted a systematic search for transdifferentiation recipes that could achieve reprogramming. The three-gene recipes identified achieved remarkable success the best of which achieve final correlations, with the target state, in the range 0.9351≤PearsonR≤0.9812. Master inter-neuron regulators are identified as SATB2 and GAD1 as well the identification of POU6F2 and ADARB2 as important intra-neuron regulators.
